# Anemia modifies the prognostic value of glycated hemoglobin in patients with diabetic chronic kidney disease

**DOI:** 10.1371/journal.pone.0199378

**Published:** 2018-06-22

**Authors:** I-Ching Kuo, Hugo You-Hsien Lin, Sheng-Wen Niu, Jia-Jung Lee, Yi-Wen Chiu, Chi-Chih Hung, Shang-Jyh Hwang, Hung-Chun Chen

**Affiliations:** 1 Department of Internal Medicine, Kaohsiung Municipal Ta-Tung Hospital, Kaohsiung Medical University, Kaohsiung, Taiwan; 2 Graduate Institute of Clinical Medicine, Kaohsiung Medical University, Kaohsiung, Taiwan; 3 Graduate Institute of Medicine, College of Medicine, Kaohsiung Medical University, Kaohsiung, Taiwan; 4 Division of Nephrology, Department of Internal Medicine, Kaohsiung Medical University Hospital, Kaohsiung Medical University, Kaohsiung, Taiwan; 5 Faculty of Renal Care, College of Medicine, Kaohsiung Medical University, Kaohsiung, Taiwan; Universita degli Studi di Perugia, ITALY

## Abstract

A common complication of chronic kidney disease (CKD), anemia can influence glycated hemoglobin (HbA1c) levels. In diabetic patients, anemia occurs earlier and with higher severity over the course of CKD stages. To elucidate the effect of hemoglobin (Hb) on the predictive value of HbA1c, we enrolled 1558 diabetic patients with stages 3–4 CKD, categorized according to baseline Hb and HbA1c quartiles. Linear regression revealed that higher HbA1c correlated significantly with higher Hb in the Hb < 10 g/dL group (β = 0.146, *P* = 0.004). A fully-adjusted Cox regression model revealed worse clinical outcomes in patients with higher HbA1c quartiles in the Hb ≥ 10 g/dL group. Hazard ratios for end-stage renal disease (ESRD), all-cause mortality, and composite endpoint (cardiovascular events and all-cause mortality) in patients with Hb ≥ 10 g/dL and the highest HbA1c quartile were 1.92 (95% confidence interval [CI], 1.17–3.15), 1.76 (95% CI, 1.02–3.03), and 1.54 (95% CI, 1.03–2.31), respectively. By contrast, HbA1c was not associated with clinical outcomes in the Hb < 10 g/dL group. In conclusion, in stages 3–4 diabetic CKD, higher HbA1c is associated with a higher risk of poor clinical outcomes in patients with Hb ≥ 10 g/dL.

## Introduction

Anemia is a common feature in patients with chronic kidney disease (CKD), and it is mainly attributable to the relative decrease in erythropoietin (EPO) production by the kidneys, absolute or functional iron deficiency, and shortened red cell survival[1]. According to the National Health and Nutrition Examination Survey[2], the prevalence of anemia increases as estimated glomerular filtration rate (eGFR) below 60 mL/min/1.73 m^2^. However, in patients with diabetic kidney disease, anemia occurs earlier[3, 4] and tends to be of greater severity stratified by CKD stages and albuminuria compared with those without diabetes[5–8]. The reimbursement of erythropoietin stimulating agents (ESA), which is strictly regulated in some countries, may affect the prevalence of anemia.

In diabetes mellitus (DM), good glycemic control, which is expressed through glycated hemoglobin (HbA1c) measurements, has been confirmed to prevent or delay the occurrence of microvascular complications. Several observational studies involving diabetic patients with stages 3–4 CKD have demonstrated that a baseline HbA1c of ≥ 9% is independently associated with a higher risk of mortality and end-stage renal disease (ESRD)[9, 10]. However, it is clarified that HbA1c levels can be altered by anemia, uremic environments, or ESA administration[11]. HbA1c levels may not optimally represent the general glycemic state in CKD populations. The results of our previous study revealed a lack of prognostic value of HbA1c levels in stage 5 CKD[10]. Accordingly, the reliance on HbA1c levels to predict prognosis in CKD patients with anemia might be doubtful. By contrast, previous studies have already suggested that protein–energy malnutrition and chronic inflammation apparently influence anemia development in patients with CKD[12, 13]; lower HbA1c levels might indicate a poor nutritional status or more severe degree of inflammation, in addition to glycemic control. Therefore, the present study attempts to assess whether anemia modifies the predictive role of HbA1c levels in patients with stages 3–4 diabetic CKD, who did not receive ESA treatment.

## Methods

### Participants and measurements

This observational study enrolled patients with CKD from integrated or traditional CKD care programs conducted in two affiliated hospitals of Kaohsiung Medical University in Southern Taiwan from November 11, 2002, to May 31, 2009. CKD was defined according to the National Kidney Foundation’s Kidney Disease Outcomes Quality Initiative[14], and the eGFR was calculated using the equation from the four-variable Modification of Diet in Renal Disease (MDRD) study. Patients were excluded if they had acute kidney injury, defined as a decrease of > 50% in the eGFR within 3 months, or if they had undergone renal replacement therapy (RRT) before their first visit. In Taiwan, National Health Insurance regulations limit ESA administration until serum creatinine is > 6 mg/dL; therefore, patients with stages 3–4 CKD did not receive ESAs. The diagnosis of type 2 DM was defined by the World Health Organization and use of either oral hypoglycemic agents or insulin[15]. 1558 patients with diabetes and stages 3–4 CKD were included. All patients periodically received follow-ups until May 31, 2010, for serial blood exams and evaluation of CKD complications. Data were obtained with the informed consent of all participants and the approval of the Kaohsiung Medical University Hospital Institutional Review Board, in accordance with the Declaration of Helsinki.

All biochemical data were recorded at the first visit (baseline). Baseline blood samples were drawn after an overnight fast, and all laboratory values, including serum creatinine, albumin, hemoglobin (Hb), blood glucose, HbA1c, total calcium, phosphate, uric acid, total cholesterol, triglyceride, C-reactive protein (CRP), and urine protein-to-creatinine ratio (UPCR), were determined through standardized methods. The eGFR was calculated using the MDRD equation, which is generally applied in the Taiwan National Database to assess CKD prevalence and dialysis initiation[16, 17]. HbA1c values were measured as clinically indicated through standard automated cation-exchange high-performance liquid chromatography. We classified participants into two groups according to their baseline Hb levels: < 10 g/dL and ≥ 10 g/dL. Each group was subsequently divided into quartiles on the basis of HbA1c values at 6.4, 7.2 and 8.3%.

All demographic information and relevant medical histories of the participants, including data on their age, sex, blood pressure (BP), stroke, cardiovascular disease (CVD), and congestive heart failure (CHF), were collected from the medical records, at the time of enrolment. Weight and height were assessed, and the body mass index (BMI) was calculated. Hypertension was diagnosed as systolic BP ≥ 140 mmHg and/or diastolic BP ≥ 90 mmHg and/or by the requirement of antihypertensive treatment. CVD was defined as clinically diagnosed myocardial infarction, heart failure, ischemic heart disease, or cerebrovascular disease.

### Clinical outcomes

The primary outcomes measures were ESRD, CV events, and all-cause mortality. ESRD was defined by a history of RRT (initiation of hemodialysis and peritoneal dialysis or renal transplantation). ESRD development was ascertained using catastrophic illness cards issued by the Bureau of National Health Insurance. Hospital records were analysed to identify CV events and the most responsible diagnoses of acute coronary syndrome (International Classification of Diseases, Ninth Revision, Clinical Modification: 410.x–412.x), acute cerebrovascular disease (430.x–438.x), and CHF (428.x) and death from the aforementioned causes (but only in patients with the occurrence of CV events after the index date). All-cause mortality was determined through death certificates and the National Death Index.

### Statistical analysis

Descriptive statistics were summarized as the frequency and percentage for categorical data and means with standard deviations (or medians with interquartile ranges) for continuous variables with approximately normal distributions. Between-groups comparisons of the baseline characteristics were achieved by ANOVA for normally distributed variables and by chi-square tests for categorical variables. Logarithmic transformation was applied for variables with a skewed distribution (cholesterol and CRP). Multiple imputation was applied for missing data in iron and ferritin. The correlation of Hb with HbA1c levels was constructed through multivariable linear regression analysis.

To determine the variables that were independently predictive of HbA1c levels, multivariable linear regression analysis was performed. To identify the effects of baseline HbA1c (stratified by Hb) on clinical outcomes, Cox proportional regression models were employed. The models were adjusted to control for the effects of related factors including age, sex, eGFR, log-transformed UPCR, CVD, mean BP, log-transformed cholesterol, log-transformed CRP, phosphorus levels, BMI, Hb, albumin, and iron. Covariates were selected on the basis of their statistical significance or clinical relevance. We also applied several sensitivity analyses to account for the effects on clinical outcomes with different classifications divided: (1) by clinical relevance of HbA1c values: < 6%, ≥ 6% to < 7%, ≥ 7% to < 9%, and ≥ 9%, and (2) by fasting glucose levels. The results are expressed as hazard ratios (HRs) with 95% confidence intervals (CIs). For all analyses, all tests were two-tailed and results with *P* < 0.05 were considered statistically significant. Statistical analyses were conducted using R 3.3.0 software (R Foundation for Statistical Computing, Vienna, Austria) and SPSS Version 21.0 for Windows (SPSS Inc., Chicago, IL, USA).

## Results

### Baseline characteristics of diabetic patients with stages 3–4 CKD, stratified by Hb and HbA1c quartiles

Baseline characteristics of the 1558 patients classified by Hb and HbA1c quartiles are shown in Table 1. This cohort had a mean age of 64.7 ± 12.8 years, mean Hb level of 11.6 ± 2.2 g/dL, mean HbA1c level of 7.6% ± 1.8%, median UPCR of 1164 (322–3297) mg/g, and mean eGFR of 33.0 ± 11.9 mL/min/1.73 m^2^. To assess the effect of anemia, the participants were divided into two groups: 411 patients with Hb < 10 g/dL and 1147 with Hb ≥ 10 g/dL. In both groups, patients with higher HbA1c levels had higher levels of total cholesterol and triglyceride (all *P* for trend < 0.05), but not a higher percentage of CVD. Additionally, in the Hb ≥ 10 g/dL group, higher HbA1c levels exhibited higher UPCR (*P* for trend < 0.05). As for the iron profile evaluating the cause of anemia in both groups, there were lower iron and higher prevalence of iron deficiency in the Hb < 10 g/dL group, compared with Hb ≥ 10 g/dL group (*P* < 0.05). There no difference in iron profile among HbA1c groups in patients with Hb < 10 g/dL.

**Table 1:** Baseline characteristics of patients with stages 3–4 CKD, stratified by Hb and HbA1c quartiles.

	All	Hemoglobin < 10 g/dl				*P*	Hemoglobin ≥ 10 g/dl				*P*
		HbA1c quartiles					HbA1c quartiles				
		Q1	Q2	Q3	Q4		Q1	Q2	Q3	Q4	
**HbA1c (%)**		< 6.4	6.4–7.2	7.2–8.3	≥ 8.3		< 6.4	6.4–7.2	7.2–8.3	≥ 8.3	
**No. of patients**	1558	136	90	91	94		256	282	322	287	
**Demographics and medical history**											
Age (years)	64.7 (12.8)	67.2 (12.9)	66.0 (12.4)	65.1 (11.6)	62.8 (13.3)	0.001	67.0 (11.9)	63.6 (14.2)	64.3 (13.3)	62.9 (11.6)	0.001
Female (n[%])	589 (37.8%)	81 (59.6%)	38 (42.2%)	50 (54.9%)	53 (56.4%)	0.073	82 (32.0%)	89 (31.6%)	107 (33.2%)	89 (31.0%)	0.944
Hypertension (n[%])	1695 (71%)	107 (78.7%)	63 (70.0%)	61 (67.0%)	62 (66.0%)	0.121	186 (72.7%)	171 (60.6%)	198 (61.5%)	177 (61.7%)	0.011
CVD (n[%])	812 (33.8%)	52 (38.2%)	36 (40.0%)	28 (30.8%)	28 (29.8%)	0.327	88 (34.4%)	86 (30.5%)	69 (21.4%)	99 (34.5%)	0.001
CHF (n[%])	406 (16.9%)	28 (20.6%)	22 (24.4%)	21 (23.1%)	16 (17.0%)	0.620	28 (10.9%)	42 (14.9%)	22 (6.8%)	41 (14.3%)	0.007
Stroke (n[%])	492 (20.5%)	35 (25.7%)	21 (23.3%)	16 (17.6%)	13 (13.8%)	0.125	68 (26.6%)	52 (18.4%)	48 (14.9%)	66 (23.0%)	0.003
BMI (m^2^/kg)	25.2 (3.9)	23.4 (3.4)	24.4 (4.8)	24.2 (3.8)	24.6 (3.9)	0.037	25.1 (3.6)	26.1 (4.0)	26.0 (4.1)	25.9 (3.7)	0.102
MAP (mmHg)	99.3 (14.0)	96.7 (14.1)	96.9 (15.1)	99.2 (14.1)	98.8 (13.8)	0.034	99.2 (13.6)	98.7 (13.1)	100.2 (14.0)	101.1 (14.8)	0.003
**Hb and iron profiles**											
Hemoglobin (g/dl)	11.6 (2.2)	8.9 (0.9)	8.9 (1.1)	8.9 (0.9)	9.1 (0.8)	0.012	12.6 (1.6)	12.7 (1.7)	12.5 (1.7)	12.4 (1.6)	0.644
Iron (mg/dl)	69.8 (27.2)	57.6 (26.3)	59.0 (30.0)	54.1 (25.6)	60.8 (30.1)	0.617	83.7 (28.3)	77.0 (26.9)	71.5 (28.5)	70.7 (29.6)	<0.001
Ferritin (ng/ml)	212 (110–387)	201 (109–454)	273 (113–481)	240 (104–467)	222 (114–413)	0.761	201 (108–345)	203 (96–352)	193 (88–331)	200 (107–387)	0.311
Iron deficiency (n[%]) ^a^	547 (46.2%)	69 (67.6%)	49 (69.0%)	33 (62.3%)	36 (58.1%)	0.577	66 (29.9%)	80 (35.7%)	109 (48.7%)	105 (46.5%)	<0.001
**Laboratory data**											
eGFR (ml/min/1.73 m^2^)	33.0 (11.9)	25.3 (8.6)	26.6 (9.5)	27.6 (10.7)	28.2 (12.3)	0.897	34.5 (11.5)	36.8 (11.8)	35.8 (11.8)	34.0 (11.5)	0.086
UPCR (mg/g)	1164 (322–3297)	1714 (711–4227)	1957 (652–5390)	2970 (977–6135)	3106 (1345–5787)	0.073	567 (250–1757)	545 (167–1532)	923 (268–2885)	1499 (452–4187)	<0.001
Albumin (g/dl)	3.8 (0.6)	3.5 (0.7)	3.5 (0.6)	3.4 (0.7)	3.4 (0.6)	0.430	4.0 (0.5)	4.0 (0.5)	3.9 (0.6)	3.7 (0.5)	0.242
Blood glucose (mg/dl)	134.8 (56.4)	105.4 (31.4)	127.6 (46.0)	141.2 (59.0)	162.5 (75.6)	<0.001	106.4 (28.3)	118.2 (41.2)	139.3 (45.7)	176.2 (71.5)	<0.001
HbA1c (%)	7.6 (1.8)	5.7 (0.5)	6.8 (0.2)	7.7 (0.3)	9.7 (1.5)	<0.001	5.8 (0.5)	6.8 (0.2)	7.7 (0.3)	10.2 (1.5)	<0.001
Total calcium (mg/dl)	9.3 (0.7)	9.0 (0.7)	8.9 (0.7)	9.1 (0.6)	9.2 (0.8)	0.001	9.3 (0.6)	9.4 (0.6)	9.3 (0.7)	9.3 (0.7)	0.003
Phosphate (mg/dl)	4.0 (0.9)	4.4 (0.7)	4.4 (1.0)	4.2 (0.9)	4.2 (0.9)	0.155	3.8 (0.8)	3.8 (0.8)	3.9 (0.9)	4.0 (0.9)	0.001
Uric acid (mg/dl)	7.8 (1.9)	7.8 (1.7)	8.1 (2.1)	7.5 (2.0)	8.2 (2.9)	<0.001	7.9 (1.9)	7.9 (1.8)	7.6 (1.7)	7.6 (1.9)	0.003
Total cholesterol (mg/dl)	193 (164–226)	177 (146–210)	177 (153–216)	187 (162–230)	201 (167–246)	<0.001	187 (164–215)	194 (164–223)	196 (164–225)	209 (174–250)	<0.001
Triglyceride (mg/dl)	140 (100–208)	115 (78–166)	119 (86–158)	150 (96–202)	157 (108–241)	<0.001	124 (92–182)	138 (98–210)	145 (103–212)	180 (118–261)	<0.001
CRP (mg/l)	1.6 (0.4–10.0)	1.6 (0.5–10.4)	4.4 (0.7–19.7)	3.0 (0.5–35.0)	1.3 (0.4–13.3)	0.184	1.0 (0.3–5.7)	1.2 (0.4–7.9)	1.5 (0.5–8.7)	2.1 (0.5–14.6)	0.017

Data expressed as the mean ± standard deviation, median (interquartile range), or count (percentage).

Abbreviations: HbA1c, glycated hemoglobin; CVD, cardiovascular disease; CHF, congestive heart failure; BMI, body mass index; MAP, mean arterial pressure; Hb, hemoglobin; eGFR, estimated glomerular filtration rate; UPCR, urine protein-to-creatinine ratio; CRP, C-reactive protein.

^a.^ Iron deficiency was defined as iron saturation <20% or ferritin < 100 ng/ml.

During the 3-year follow-up period, 132 (32.1%) and 180 (15.7%) incident cases of ESRD were observed among the patients with Hb < 10 and Hb ≥ 10 g/dL, respectively, in Table 2. In the Hb ≥ 10 g/dL group, patients with higher HbA1c levels had higher rates of ESRD. There were 110 (26.8%) and 161 (14.0%) incident cases of all-cause mortality among the patients with Hb < 10 and Hb ≥ 10 g/dL, respectively. Furthermore, higher incidence rates of all-cause mortality and composite outcomes were associated with higher HbA1c levels in the Hb ≥ 10 g/dL group.

**Table 2:** Clinical outcomes of patients with stages 3–4 CKD, stratified by Hb and HbA1c quartiles.

	All	Hemoglobin < 10 g/dl				*P*	Hemoglobin ≥ 10 g/dl				*P*
		HbA1c quartiles					HbA1c quartiles				
		Q1	Q2	Q3	Q4		Q1	Q2	Q3	Q4	
HbA1c (%)		< 6.4	6.4–7.2	7.2–8.3	≥ 8.3		< 6.4	6.4–7.2	7.2–8.3	≥ 8.3	
No. of patients	1558	136	90	91	94		256	282	322	287	
Follow-up days	1029 (607–1642)	837 (532–1597)	875 (520–1512)	962 (507–1551)	1025 (554–1765)	0.595	1028 (637–1654)	1061 (587–1633)	1051 (581–1695)	1171 (763–1654)	0.216
Annual eGFR decline (ml/min/1.73 m^2^/year)	-3.0 (-7.4 to 0.1)	-5.4 (-9.4 to -1.6)	-5.1 (-10.3 to -0.9)	-5.8 (-13.2 to -1.6)	-5.0 (-10.2 to -1.0)	0.767	-1.5 (-4.5 to 0.7)	-1.3 (-4.4 to 1.4)	-2.4 (-6.4 to 0.0)	-4.0 (-9.6 to -0.4)	<0.001
Rapid eGFR decline ^a^	563 (36.1%)	72 (52.9%)	45 (50.0%)	48 (52.7%)	47 (50.0%)	0.953	60 (23.4%)	65 (23.0%)	98 (30.4%)	128 (44.6%)	<0.001
ESRD	312 (20.0%)	44 (32.4%)	27 (30.0%)	36 (39.6%)	25 (26.6%)	<0.001	32 (12.5%)	25 (8.9%)	48 (14.9%)	75 (26.1%)	<0.001
All-cause mortality	271 (17.4%)	33 (24.3%)	30 (33.3%)	26 (28.6%)	21 (22.3%)	0.320	23 (9.0%)	38 (13.5%)	42 (13.0%)	58 (20.2%)	0.002
CV event + all-cause mortality	390 (25.0%)	48 (39.3%)	35 (38.9%)	36 (39.6%)	31 (33.0%)	0.653	43 (16.8%)	46 (16.3%)	59 (18.4%)	92 (32.1%)	<0.001

Abbreviations: ESRD, end-stage renal disease; CV, cardiovascular.

^a^ Annual eGFR decline more than −5 mL/min/1.73 m^2^/year

### Variables associated with HbA1c levels

**Fig 1:**
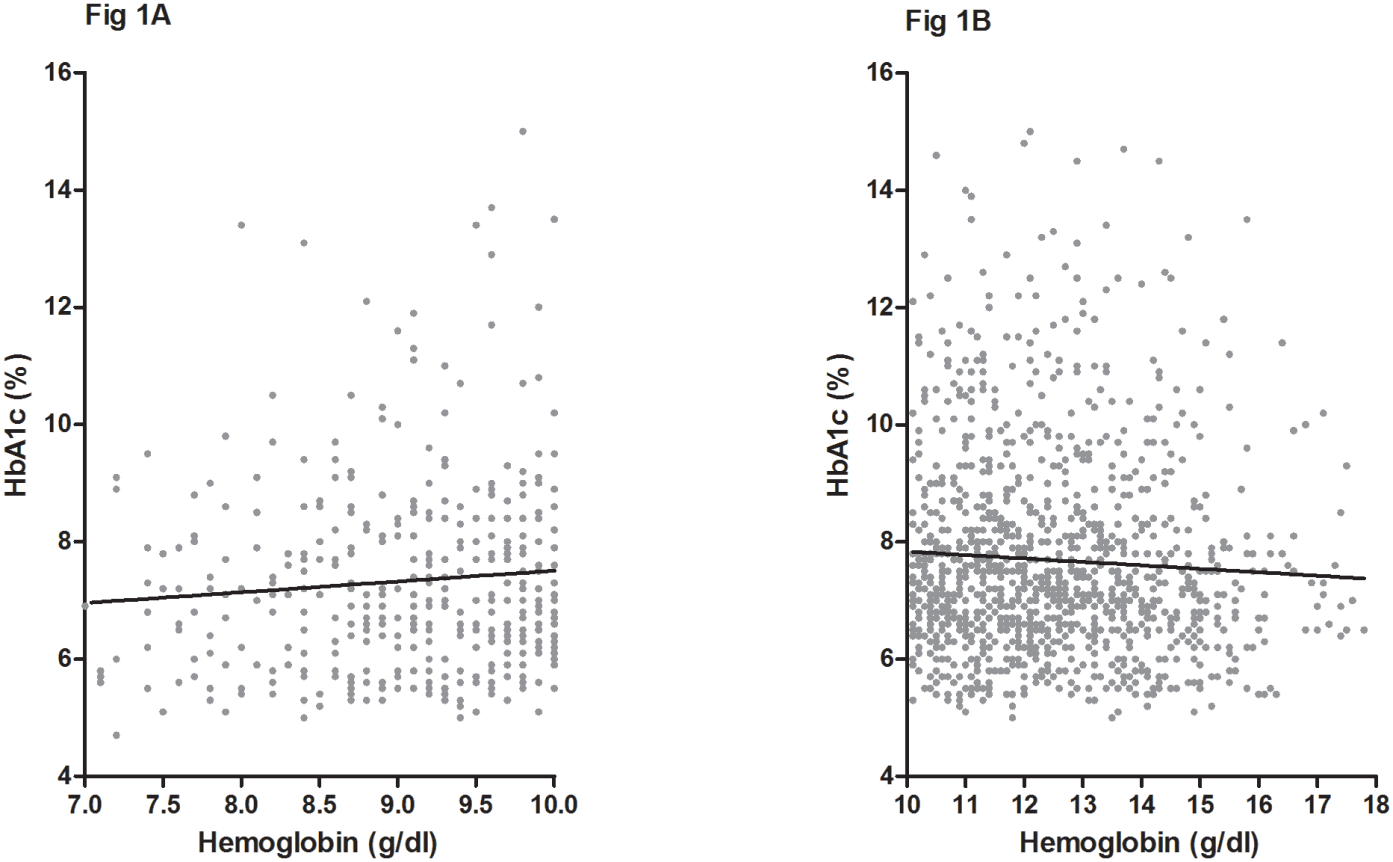
Regression diagram for HbA1c and Hb levels among subjects with (A) Hb < 10 g/dL and (B) Hb ≥ 10 g/dL.

Table 3 shows the relevant covariates of the HbA1c levels in multivariable linear regression. HbA1c levels correlated with age, eGFR, log UPCR, Hb levels, and log cholesterol. However, HbA1c levels correlated significantly with Hb levels only in the Hb < 10 g/dL group (95% CI, 0.047–0.24; *P* = 0.004) but not in the Hb ≥ 10 g/dL group (95% CI, −0.054–0.082; *P* = 0.689; Table 3 and Fig 1).

**Table 3:** Multivariable linear regression of HbA1c levels.

	All		
Variables	β	95% CI	*p*-value
Age (years)	-0.009	-0.014 to -0.003	0.002
Male vs. female	0.029	-0.116 to 0.174	0.693
CVD	0.030	-0.113 to 0.173	0.680
BMI (kg/m^2^)	0.002	-0.015 to 0.020	0.795
MAP (mmHg)	-0.002	-0.007 to 0.003	0.421
eGFR (ml/min/1.73 m^2^)	0.012	0.005 to 0.018	<0.001
Log UPCR	0.308	0.146 to 0.469	<0.001
Albumin (g/dl)	-0.210	-0.343 to -0.077	0.002
Hemoglobin (g/dl)			
In total population	0.099	0.058 to 0.141	<0.001
In Hb ≥ 10 g/dl*	0.014	-0.054 to 0.082	0.689
In Hb < 10 g/dl*	0.146	0.047 to 0.245	0.004
Log cholesterol	2.098	1.524 to 2.672	<0.001
Log CRP	0.059	-0.011 to 0.130	0.097
Phosphate (mg/dl)	-0.063	-0.133 to 0.006	0.072

Abbreviations: Hb, hemoglobin; CVD, cardiovascular disease; BMI, body mass index; MAP, mean arterial pressure; eGFR, estimated glomerular filtration rate; UPCR, urine protein-to-creatinine ratio; CRP, C-reactive protein.

* Segmental linear regression with the same variables

*P* < 0.05 indicates a significant association with HbA1c levels.

### Relation between HbA1c quartiles and clinical outcomes

In the Hb ≥ 10 g/dL group (Table 4), a fully adjusted multivariable Cox regression model revealed an increased risk of RRT in the third quartile (HR = 1.65, 95% CI, 0.99–2.76; *P* = 0.057), and the highest quartile (HR = 1.92, 95% CI, 1.17–3.15; *P* = 0.010) (*P* for trend = 0.042) compared with the first quartile subgroup. In addition, a trend of increased risks of composite outcomes (CV events combined with all-cause mortality) was associated with the third quartile (HR = 1.51, 95% CI, 0.98–2.32; *P* = 0.059), and the highest quartile (HR = 1.54, 95% CI, 1.03–2.31; *P* = 0.036) (*P* for trend = 0.171). Similarly, significantly increased risks of all-cause mortality were related to the second quartile (HR = 2.17, 95% CI, 1.23–3.84; *P* = 0.012) and the highest quartile (HR = 1.76, 95% CI, 1.02–3.03; *P* = 0.043) (*P* for trend = 0.063). However, in the Hb < 10 g/dL group (Table 4), the fully adjusted Cox regression model did not indicate a significant risk trend for clinical outcomes for the different HbA1c quartiles compared with the first quartile subgroup.

**Table 4:** Risk of outcomes among subjects with Hb < 10 g/dL and Hb ≥ 10 g/dL, stratified by HbA1c quartiles.

	All				Hemoglobin < 10 g/dl				Hemoglobin ≥ 10 g/dl			
	HbA1c quartiles				HbA1c quartiles				HbA1c quartiles			
	Q1	Q2	Q3	Q4	Q1	Q2	Q3	Q4	Q1	Q2	Q3	Q4
**HR (95% CI) for RRT**												
Unadjusted	1	0.68 (0.46–1.00)*	1.13 (0.81–1.58)	1.28 (0.93–1.77)	1	0.80 (0.47–1.34)	1.12 (0.69–1.79)	0.59 (0.35–1.00)	1	0.69 (0.38–1.23)	1.42 (0.88–2.30)	2.25 (1.44–3.54)*
Adjusted ^a^	1	1.05 (0.71–1.57)	1.37 (0.96–1.93)	1.34 (0.95–1.87)	1	1.24 (0.71–2.17)	1.32 (0.80–2.20)	0.79 (0.45–1.36)	1	1.14 (0.62–2.10)	1.65 (0.99–2.76)	1.92 (1.17–3.15)*
**HR (95% CI) for all-cause mortality**												
Unadjusted	1	1.43 (0.98–2.11)	1.23 (0.83–1.81)	1.51 (1.04–2.19)*	1	1.51 (0.88–2.60)	1.07 (0.59–1.94)	0.96 (0.53–1.71)	1	1.69 (0.96–2.96)	1.68 (0.97–2.91)	2.34 (1.38–3.96)*
Adjusted ^a^	1	1.79 (1.21–2.65)*	1.30 (0.87–1.93)	1.49 (1.01–2.18)*	1	1.62 (0.92–2.85)	0.94 (0.50–1.76)	1.04 (0.56–1.91)	1	2.17 (1.23–3.84)*	1.72 (0.98–3.02)	1.76 (1.02–3.03)*
**HR (95% CI) for CV event + all-cause mortality**												
Unadjusted	1	0.96 (0.70–1.33)	1.07 (0.78–1.45)	1.45 (1.08–1.95)*	1	1.13 (0.70–1.81)	1.05 (0.65–1.69)	0.89 (0.55–1.44)	1	1.01 (0.65–1.58)	1.27 (0.84–1.93)	2.07 (1.40–3.06)*
Adjusted ^a^	1	1.26 (0.91–1.76)	1.28 (0.93–1.75)	1.40 (1.03–1.89)*	1	1.20 (0.73–1.97)	1.05 (0.64–1.73)	1.01 (0.61–1.67)	1	1.29 (0.82–2.03)	1.51 (0.98–2.32)	1.54 (1.03–2.31)*

^a^ The Cox proportional hazard model was adjusted for age, sex, estimated glomerular filtration rate, log (urine protein-to-creatinine ratio), cardiovascular disease, hypertension, mean blood pressure, hemoglobin, albumin, log (cholesterol), log (C-reactive protein), phosphorus, body mass index and iron.

Abbreviations: RRT, renal replacement therapy

* *p* < 0.05 indicates significant differences compared with the reference group

To explore the independent effects of variables on RRT, we conducted sequential models to examine changes in parameter estimates. Cox regression model with 4 incremental levels of covariate adjustment yielded similar results to the primary analysis and is shown in S1 Table.

### Sensitivity analyses

In the Hb ≥ 10 g/dL group, sensitivity analysis reclassifying patients into subgroups by clinical relevance showed similar tendency towards an increased HR for RRT, with HbA1c levels of ≥ 6% to < 7%, ≥ 7% to < 9%, and ≥ 9%, having HRs of 1.83 (95% CI, 0.92–3.65), 2.43 (95% CI, 1.26–4.68), and 3.06 (95% CI, 1.56–5.99) (*P* for trend = 0.008), respectively, when compared with HbA1c < 6% (S2 Table). Similarly, increased risks of composite outcomes (CV events combined with all-cause mortality) were associated with HbA1c levels with HRs of 1.41 (95% CI, 0.88–2.26) for ≥ 7% to < 9%, and 1.66 (95% CI, 1.02–2.70) for ≥ 9%, respectively (*P* for trend = 0.194). Also, increased risks of all-cause mortality were associated with HbA1c levels with HRs of 1.64 (95% CI, 0.92–2.95) for ≥ 7% to < 9%, and 1.88 (95% CI, 1.00–3.52) for ≥ 9%, respectively (*P* for trend = 0.079). By contrast, the association was not observed in patients with Hb < 10 g/dL group (S2 Table).

Furthermore, to clarify the direct effect of glucose control on RRT, we reclassified the patients into four subgroups according to their mean plasma glucose levels, with glucose levels of 125, 155, and 210 mg/dL corresponding to HbA1c levels of 6%, 7%, and 9% (S3 Table). An increased risk of RRT was associated with mean plasma glucose levels of 125–155 mg/dL (HR = 1.21, 95% CI, 0.62–2.36), 155–210 mg/dL (HR = 1.07, 95% CI, 0.55–2.07), and ≥ 210 mg/dL (HR = 2.32, 95% CI, 1.04–5.19) (*P* for trend = 0.164).

## Discussion

In diabetic patients with stages 3–4 CKD, we found that higher HbA1c levels are associated with a trend of increased risks for RRT, all-cause mortality and composite outcomes. In the Hb ≥ 10 g/dL group, the magnitudes of association were prominent and statistically significant among those with the highest HbA1c quartile compared with those with the lowest quartile. In patients with Hb < 10 g/dL, HbA1c levels were not associated with clinical outcomes; however, mean plasma glucose levels could be more useful in predicting RRT risk.

Anemia occurs commonly in patients with CKD as serum Hb levels correlate almost linearly with eGFR[18]. In diabetic patients, the prevalence of anemia is higher even in the absence of nephropathy, and DM has been indicated as an independent determinant of Hb levels[3, 19]. Several mechanisms have been described for the link between anemia and diabetes: (1) reduced erythropoietin production attributed to splanchnic sympathetic denervation of the kidneys resulting from diabetic autonomic neuropathy[20]; (2) impairment of the hypoxia-sensing mechanism secondary to vascular and tubulointerstitial lesions[21, 22]; (3) chronic systemic inflammation contributing to erythropoietin hyporesponsiveness and functional iron deficiency through increased hepcidin levels, which are independent of uremic toxin retention[23, 24]; (4) increased urinary excretion of transferrin and erythropoietin as a result of nonselective proteinuria[25]; and (5) renin–angiotensin system blockade partly impeding the physiologic erythropoietic effects of angiotensin II[26]. Several observational studies, including our cohort study, have recognized that patients with diabetic CKD present higher prevalence of anemia than that in non-diabetic counterparts, across all ranges of kidney function (CKD stages 1–5)[4, 23].

HbA1c levels are widely used to assess the degree of glycemic control and risk prediction of future vascular complications, in addition to being a diagnostic marker of diabetes. However, the underlying challenges associated with the predictive ability of HbA1c persist in CKD environments, including the accurate reflection of glycemic control and the related outcomes[11]. In addition to glucose, HbA1c levels may be falsely elevated or decreased in CKD because a uremic environment shortens the red blood cell (RBC) lifespan, and carbamylated Hb formed in the presence of high urea interferes with glycosylation of Hb[27, 28]. Many factors present in CKD have an impact on RBC turnover, including fragile RBCs, ESA administration, iron deficiency anemia, and transfusion therapy[28–30]. Prior published reports have supported this notion. Freedman et al. reported an inverse correlation between eGFR and the glucose/HbA1c ratio in diabetic patients with stages 3–4 CKD[31]. Kim et al. also demonstrated that among diabetic patients with pre-dialysis CKD, glucose/HbA1c and glycated albumin/HbA1c ratios correlated inversely with eGFR, whereas the glucose/glycated albumin ratio did not[32]. Considering the effect of Hb, Agarwal et al. reported that among 128 patients with DM and CKD, a decline in HbA1c correlated with advancing CKD stages, but the statistical significance was removed after adjustment for Hb[33]. Taken together, HbA1c levels appear to falsely decrease with the declining eGFR in patients with DM and advanced CKD. In our cohort, we confirmed that eGFR significantly affected HbA1c independently in all stages 3–4 CKD patients through the regression model. Moreover, it was established that the positive correlation between Hb and HbA1c levels only occurred in the Hb < 10 g/dL group.

HbA1c levels might be used to predict clinical outcomes more accurately in CKD patients without anemia. Se Won Oh et al. explored that 799 diabetic patients with eGFR < 60 mL/min/1.73m^2^ showed increased risks of ESRD as HbA1c levels increased from 6.5%[34]. A large observational study conducted by Shurraw et al. on 23 000 patients with DM and eGFR < 60 mL/min/1.73m^2^ demonstrated that baseline HbA1c > 7% was associated with increased risks of all-cause mortality, myocardial infarction, and ESRD[9]. To the best of our knowledge, although HbA1c levels could be affected by Hb variability, none of these studies have examined this effect. In our diabetic patients with stages 3–4 CKD and Hb < 10 g/dL, we observed that higher HbA1c levels were not predictive of inferior clinical outcomes, and similar results were observed with different HbA1c classifications. By contrast, higher average fasting glucose levels remained associated with higher RRT risks. In this regard, we speculate that the use of direct glucose monitors or alternative markers other than HbA1c levels for evaluating glycemic state and predicting clinical outcomes might eliminate the confounding effect as kidney function declines accompanied by Hb drops. Recently, glycated albumin which is not affected by Hb appears to be a more appropriate representation of short-term glycemic control and glucose fluctuations compared with HbA1c in CKD patients[11]. Besides, cumulative evidence supports the predictive role of glycated albumin in diabetic complications [35–38]. This might provide a solution to improve the accuracy as a marker of glycemic control in diabetic CKD populations.

Anemia is also regarded as an index of chronic inflammation and poor nutrition[12, 13]. Several studies have provided the speculation that in populations with a high prevalence of inflammation and malnutrition, such as advanced CKD, HbA1c levels alone could be less predictive of clinical outcomes. Our previous analysis demonstrated that HbA1c ≥ 9% predicts ESRD and composite outcomes in CKD stages 3–4 patients but not in stage 5 patients[10]. Kalantar et al. demonstrated that in diabetic patients undergoing maintenance hemodialysis and with Hb < 11 g/dL, higher HbA1c levels were not associated with increased risks of mortality, as seen in nonanemic patients[39]. Moreover, from the post hoc analysis of the Trial to Reduce Cardiovascular Events With Aranesp Therapy in diabetic CKD patients with anemia, higher baseline CRP levels, known as inflammation biomarkers, were associated with a greater risk of future ESRD, whereas higher HbA1c levels did not present the association[40]. In our current study, in which diabetic patients with stages 3–4 CKD and without ESA therapy were recruited, one possibility might be that a lower HbA1c level identifies individuals with lower Hb instead of simply reflecting glycemic control, particularly among patients with Hb < 10 g/dL. Even though the hemoglobin adjustment was done, HbA1c was still not predictive, which might imply other mechanisms.

This study had some limitations. First, we relied on the baseline measurement of HbA1c values for analysis rather than mean values. However, because of the legacy effect of HbA1c and the influence of eGFR on HbA1c levels, it is reasonable to use baseline HbA1c as an indicator, when CKD stages were classified at simultaneously. Second, we did not have explicit surrogate nutrition markers. Therefore, residual confounding based on variables related to malnutrition may remain. Third, our laboratory measurements of serum glucose levels were performed in a fasting state. We did not take postprandial glucose levels into account; however, postprandial hyperglycemia resulting from insulin resistance may affect HbA1c levels in advanced CKD[41]. Fourth, although HbA1c values obtained with immunoassays were unaffected by carbamylation in uremic environments, we were constrained to use automated cation-exchange high-performance liquid chromatography as the HbA1c assessment method in our study. Finally, the sample size of patients with Hb < 10 g/dl was only half as many patients with Hb > 10 g/dl, which might partially explain the lack of statistically significant risks.

In conclusion, among patients with stage 3–4 diabetic CKD, higher baseline HbA1c levels correlated with higher risks for ESRD, all-cause mortality, and composite outcomes (CV events and all-cause mortality) in patients with Hb ≥ 10 g/dL, whereas the association did not exist in those with Hb < 10 g/dL. Additionally, the association reached statistical significance in the highest HbA1c quartile. Further research is required to determine alternative markers (glycated albumin and continuous glucose monitoring) that might be affected to a smaller extent in CKD conditions, and the clinical target ranges at which glycemic control can reduce outcome risks in anemic CKD.

## Supporting information

S1 Table Risk of RRT among subjects with Hb < 10 g/dL and Hb ≥ 10 g/dL, stratified by HbA1c quartiles in different models. (DOCX)

S2 Table Risk of outcomes among subjects with Hb < 10 g/dL and Hb ≥ 10 g/dL, stratified by HbA1c levels. (DOCX)

S3 Table Risk of RRT among subjects with Hb < 10 g/dL, stratified by blood glucose level. (DOCX)
