# Supplementary material for: Anemia modifies the prognostic value of glycated hemoglobin in patients with diabetic chronic kidney disease
Source: PLoS One. 2018 Jun 22;13(6):e0199378. doi: 10.1371/journal.pone.0199378 (PMC6014665; doi:10.1371/journal.pone.0199378)
Supplement: S1 Table — (DOCX) [file pone.0199378.s001.docx]

**S1 Table.** Risk of RRT among subjects with Hb < 10 g/dL and Hb ≥ 10 g/dL, stratified by HbA1c quartiles in different models

|  | **All** | | | | **Hemoglobin < 10 g/dl** | | | | **Hemoglobin** ≥ **10 g/dl** | | | | |
| --- | --- | --- | --- | --- | --- | --- | --- | --- | --- | --- | --- | --- | --- |
|  | **HbA1c level (%)** | | | | **HbA1c level (%)** | | | | **HbA1c level (%)** | | | | |
|  | **Q1** | **Q2** | **Q3** | **Q4** | **Q1** | **Q2** | **Q3** | **Q4** | **Q1** | **Q2** | **Q3** | **Q4** | |
| **HR (95% CI) for RRT** | | | | | | | | | | | | |  |
| **Unadjusted** | 1 | 0.68 (0.46-1.00)* | 1.13 (0.81-1.58) | 1.28 (0.93-1.77) | 1 | 0.80 (0.47-1.34) | 1.12 (0.69-1.79) | 0.59 (0.35-1.00) | 1 | 0.69 (0.38-1.23) | 1.42 (0.88-2.30) | 2.25 (1.44-3.54)* | |
| **Model 1** | 1 | 0.93 (0.63-1.38) | 1.10 (0.78-1.55) | 1.21 (0.87-1.69) | 1 | 0.92 (0.54-1.57) | 1.12 (0.69-1.82) | 0.72 (0.42-1.23) | 1 | 1.04 (0.58-1.89) | 1.26 (0.78-2.06) | 1.78 (1.12-2.83)* | |
| **Model 2** | 1 | 0.92 (0.62-1.37) | 1.10 (0.78-1.55) | 1.21 (0.87-1.68) | 1 | 0.90 (0.52-1.54) | 1.08 (0.66-1.76) | 0.69 (0.40-1.18) | 1 | 1.05 (0.58-1.90) | 1.28 (0.78-2.10) | 1.78 (1.12-2.84)* | |
| **Model 3** | 1 | 1.06 (0.71-1.58) | 1.33 (0.94-1.89) | 1.28 (0.91-1.79) | 1 | 1.14 (0.66-1.99) | 1.26 (0.76-2.09) | 0.72 (0.42-1.24) | 1 | 1.14 (0.62-2.08) | 1.58 (0.95-2.64) | 1.84 (1.13-3.01)* | |
| **Model 4** | 1 | 1.05 (0.71-1.57) | 1.37 (0.96-1.93) | 1.34 (0.95-1.87) | 1 | 1.24 (0.71-2.17) | 1.32 (0.80-2.20) | 0.79 (0.45-1.36) | 1 | 1.14 (0.62-2.10) | 1.65 (0.99-2.76) | 1.92 (1.17-3.15)* | |

Model 1 was adjusted for age, sex, estimated glomerular filtration rate, and log (urine protein-to-creatinine ratio).

Model 2 was adjusted for cardiovascular disease, hypertension and mean blood pressure.

Model 3 was adjusted for albumin, log (cholesterol), log (C-reactive protein), phosphorus, body mass index and iron.

Model 4 was adjusted for hemoglobin.

* *p* < 0.05 indicates significant differences compared with the reference group
