# Supplementary material for: Anemia modifies the prognostic value of glycated hemoglobin in patients with diabetic chronic kidney disease
Source: PLoS One. 2018 Jun 22;13(6):e0199378. doi: 10.1371/journal.pone.0199378 (PMC6014665; doi:10.1371/journal.pone.0199378)
Supplement: S2 Table — (DOCX) [file pone.0199378.s002.docx]

**S2 Table**. Risk of outcomes among subjects with Hb < 10 g/dL and Hb ≥ 10 g/dL, stratified by HbA1c levels

|  | **All** | | | | **Hemoglobin < 10 g/dl** | | | | **Hemoglobin** ≥ **10 g/dl** | | | | |  |
| --- | --- | --- | --- | --- | --- | --- | --- | --- | --- | --- | --- | --- | --- | --- |
|  | **HbA1c level (%)** | | | | **HbA1c level (%)** | | | | **HbA1c level (%)** | | | | |  |
|  | **< 6** | **6-7** | **7-9** | ≥ **9** | **< 6** | **6-7** | **7-9** | ≥ **9** | **< 6** | **6-7** | **7-9** | ≥ **9** | |  |
| **HR (95% CI) for RRT** | | | | | | | | | | | | |  |  |
| Unadjusted | 1 | 0.76 (0.51-1.13) | 1.12 (0.79-1.60) | 1.30 (0.89-1.91) | 1 | 0.75 (0.45-1.26) | 0.88 (0.55-1.41) | 0.55 (0.28-1.06) | 1 | 1.28 (0.67-2.46) | 2.06 (1.13-3.75)* | 3.38 (1.83-6.23)* | |  |
| Adjusted ^a^ | 1 | 1.28 (0.85-1.94) | 1.46 (1.00-2.14)* | 1.62 (1.08-2.45)* | 1 | 1.32 (0.75-2.31) | 1.19 (0.72-1.98) | 0.73 (0.36-1.46) | 1 | 1.83 (0.92-3.65) | 2.43 (1.26-4.68)* | 3.06 (1.56-5.99)* | |  |
| **HR (95% CI) for all-cause mortality** | | | | | | | | | | | | | | |
| Unadjusted | 1 | 1.11 (0.74-1.68) | 1.21 (0.82-1.79) | 1.22 (0.79-1.88) | 1 | 1.23 (0.70-2.16) | 0.95 (0.54-1.67) | 0.87 (0.42-1.82) | 1 | 1.21  (0.65-2.26) | 1.72 (0.96-3.08) | 1.93 (1.04-3.59)* | |  |
| Adjusted ^a^ | 1 | 1.21 (0.80-1.83) | 1.32 (0.89-1.96) | 1.43 (0.92-2.23) | 1 | 1.15 (0.65-2.03) | 0.92 (0.52-1.64) | 1.18 (0.55-2.51) | 1 | 1.34 (0.72-2.50) | 1.64 (0.92-2.95) | 1.88 (1.00-3.52)* | |  |
| **HR (95% CI) for CV event + all-cause mortality** | | | | | | | | | | | | | | |
| Unadjusted | 1 | 0.96 (0.68-1.34) | 1.10 (0.80-1.50) | 1.23 (0.87-1.75) | 1 | 1.08 (0.67-1.75) | 1.04 (0.66-1.65) | 0.62 (0.32-1.22) | 1 | 1.02 (0.62-1.66) | 1.41 (0.89-2.22) | 2.23 (1.39-3.58)* | |  |
| Adjusted ^a^ | 1 | 1.19 (0.84-1.69) | 1.29 (0.93-1.79) | 1.48 (1.03-2.14)* | 1 | 1.25 (0.76-2.06) | 1.11 (0.69-1.80) | 0.85 (0.42-1.71) | 1 | 1.12 (0.68-1.85) | 1.41 (0.88-2.26) | 1.66 (1.02-2.70)* | |  |

^a^ The Cox proportional hazard model was adjusted for age, sex, estimated glomerular filtration rate, log (urine protein-to-creatinine ratio), cardiovascular disease, hypertension, mean blood pressure, hemoglobin, albumin, log (cholesterol), log (C-reactive protein), phosphorus, body mass index and iron.

* *p* < 0.05 indicates significant differences compared with the reference group
