# Supplementary material for: Anemia modifies the prognostic value of glycated hemoglobin in patients with diabetic chronic kidney disease
Source: PLoS One. 2018 Jun 22;13(6):e0199378. doi: 10.1371/journal.pone.0199378 (PMC6014665; doi:10.1371/journal.pone.0199378)
Supplement: S3 Table — (DOCX) [file pone.0199378.s003.docx]

**S3 Table.** Risk of RRT among subjects with Hb < 10 g/dL, stratified by blood glucose level

|  | **Hemoglobin < 10 g/dl** | | | |  |
| --- | --- | --- | --- | --- | --- |
|  | **Glucose level (mg/dl)** | | | |  |
|  | **<125** | **125-155** | **155-210** | **>210** | |
| **HR for RRT** |  |  |  |  | |
| Unadjusted | 1 | 0.81 (0.44-1.49) | 1.55 (0.85-2.83) | 2.25 (1.10-4.60)* | |
| Fully adjusted | 1 | 1.21 (0.62-2.36) | 1.07 (0.55-2.07) | 2.32 (1.04-5.19)* | |

^a^ The Cox proportional hazard model was adjusted for age, sex, estimated glomerular filtration rate, log (urine protein-to-creatinine ratio), cardiovascular disease, hypertension, mean blood pressure, hemoglobin, albumin, log (cholesterol), log (C-reactive protein), phosphorus, body mass index and iron.

* *p* < 0.05 indicates significant differences compared with the reference group
